# Specific Gray Matter Volume Changes of the Brain in Unipolar and Bipolar Depression

**DOI:** 10.3389/fnhum.2020.592419

**Published:** 2021-01-11

**Authors:** Junyan Wang, Penghong Liu, Aixia Zhang, Chunxia Yang, Sha Liu, Jizhi Wang, Yong Xu, Ning Sun

**Affiliations:** ^1^Department of Psychiatry, First Hospital of Shanxi Medical University, Taiyuan, China; ^2^Laboratory of Artificial Intelligence Assisted Diagnosis and Treatment for Mental Disorder, First Hospital of Shanxi Medical University, Taiyuan, China; ^3^Department of Mental Health, Shanxi Medical University, Taiyuan, China

**Keywords:** bipolar disorder, unipolar depression, gray matter volume, voxel-based morphology, structural MRI

## Abstract

To identify the common and specific structural basis of bipolar depression (BD) and unipolar depression (UD) is crucial for clinical diagnosis. In this study, a total of 85 participants, including 22 BD patients, 36 UD patients, and 27 healthy controls, were enrolled. A voxel-based morphology method was used to identify the common and specific changes of the gray matter volume (GMV) to determine the structural basis. Significant differences in GMV were found among the three groups. Compared with healthy controls, UD patients showed decreased GMV in the orbital part of the left inferior frontal gyrus, whereas BD patients showed decreased GMV in the orbital part of the left middle frontal gyrus. Compared with BD, UD patients have increased GMV in the left supramarginal gyrus and middle temporal gyrus. Our results revealed different structural changes in UD and BD patients suggesting BD and UD have different neurophysiological underpinnings. Our study contributes toward the biological determination of morphometric changes, which could help to discriminate between UD and BD.

## Introduction

Bipolar and unipolar depression (BD and UD) are leading causes of disability worldwide in view of the often recurrent pattern of illness, which significantly impinges on the quality of life of affected individuals (Delvecchio et al., 2012). UD and BD are both characterized by depressive symptoms, lack of interest, and loss of pleasure. Previous studies have found that 7–52% of UD patients with an initial diagnosis of BD and 0.5–1% of UD patients are converted to BD each year (Jiang et al., 2020). Until now, mental illness is diagnosed primarily through a careful assessment of behavior and subjective reports of abnormal experiences to categorize patients (Phillips and Kupfer, 2013). This means that it is difficult for clinicians to distinguish the two disorders based solely on clinical manifestations (Chen et al., 2018). The high rate of misdiagnosis can lead to a poor prognosis (Yu et al., 2020). Many researchers are seeking objective and reliable biomarkers that can differentiate between UD and BD patients. The development of neuroimaging techniques provides an opportunity to identify UD and BD and to deepen our understanding of the underlying mechanisms of affective disorders.

Neuroimaging techniques have received growing attention for studying brain alterations in affective disorders, which are associated with structural and functional abnormalities of the brain (Bremner et al., 2002; Wu et al., 2016, 2017; Wang et al., 2018, 2019b, 2020; Andreou and Borgwardt, 2020). Magnetic resonance imaging (MRI) is a non-invasive method to characterize the brain structure and function *in vivo* (Abe et al., 2010; Takamura and Hanakawa, 2017; Wang et al., 2019a). Voxel-based morphometry (VBM) is a widely used method to quantitatively detect the density and volume of brain tissue at the voxel-based level, which can reflect the differences in brain tissue composition and characteristics of individuals with BD and UD (Arnone et al., 2012, 2016, 2018; Selvaraj et al., 2012). Several studies using VBM have revealed alterations in the gray matter volume (GMV) in various brain regions in UD and BD patients compared with healthy controls (HCs) (Kandilarova et al., 2019). For example, there is reduced GMV in the prefrontal cortex (PFC), anterior cingulate cortex (ACC), hippocampus (Han et al., 2019), and amygdala (Wang et al., 2017; Calvo et al., 2019) in patients with major depressive disorder. In contrast, GMV is reduced primarily in the PFC, anterior cingulate cortex, insular cortex (Bora et al., 2010), temporal regions (Farrow et al., 2005), and orbitofrontal cortex (Eker et al., 2014) in BD patients. A meta-analysis of VBM study on UD and BD reported that GMV in the left parahippocampal gyrus and right dorsolateral PFC was significantly greater in UD patients than BD patients (Wise et al., 2017). In addition, compared with UD patients, BD patients showed reduced GMV in the right inferior frontal gyrus, middle cingulate gyrus, hippocampus, and amygdala (Bai et al., 2020). Although the previous studies have reported the structural changes in BD and UD patients, the common and specific changes in GMV in the same study remain unclear.

In this study, we recruited medication-free patients with UD and BD disorders, currently experiencing a major depressive episode. The study aimed to investigate morphometric changes in these two conditions in view of the paucity of studies directly comparing UD with BD in the same mood state and the difficulty in recruiting medication-free individuals with mood disorders (Wise et al., 2016a). Based on the findings of previous studies, we hypothesized that both BD and UD patients might show decreased GMV in PFC compared with HCs, and BD patients show decreased GMV in temporal and parietal lobes compared with UD.

## Materials and Methods

### Participants

The Ethical Committee of the First Hospital of Shanxi Medical University (20091217), Shanxi, China, approved the study. A full written and verbal explanation of the study was provided before enrollment, and each participant was provided written consent. We recruited the first-episode patients, including 22 type I BD patients and 36 UD patients from the First Hospital of Shanxi Medical University between December 2018 and July 2019. BD participants were included if they were medication-free and presented for the first time with a depressive episode and having experienced a previous manic episode, met criteria for bipolar disorder according to the Diagnostic and Statistical Manual of Mental Disorders, Fourth Edition (DSM-IV). The UD patients were included if they were medication-free and had never experienced a previous manic episode based on the diagnosis criterion of DSM-IV. The 27 HCs were recruited by advertisement. All of the participants were right-handed, according to their accounts. Two experienced psychiatrists diagnosed BD and UD according to the Structured Clinical Interview for DSM-IV criteria. The reliability coefficient of the two assessors is 0.935, and the reliability coefficient for each subitem ranges from 0.85 to 1. All the HCs were screened using the Structured Clinical Interview for DSM-IV Non-Patient Edition to confirm the lifetime absence of illness by a clinical doctor (AZ) who is one of the coauthors. For each patient, the depressive states in both BD and UD participants were evaluated based on the 24-item Hamilton Rating Scale for Depression (HAMD-24) (Williams, 1988). The anxiety states were evaluated based on the Hamilton Anxiety Scale (HAMA) (Hamilton, 1959), and the manic states were evaluated based on the Young Mania Rating Scale (YMRS) (Young et al., 1978). Inclusion criteria for BD and UD patients were as follows: (1) aged from 18 to 55 years old; (2) UD and BD patients were a total HAMD-24 score > 20, and BD patients were a total YMRS score < 7; (3) without other Axis-I and Axis-II psychiatric disorders (except for UD, BD, and anxiety disorders); (4) without a history of organic brain disorders, neurological disorders, cardiovascular diseases, alcohol/substance abuse or dependence, pregnancy, or any physical illness; (5) no electroconvulsive therapy before participating in the study; (6) UD had no family history of BD; and (7) qualified to undergo an MRI. All patients were either medication-naïve or had not been taking medication for at least 6 months at the scan time. Among all the patients, two MD patients were self-administered zopiclone 6 months ago, which has been discontinued for 6 months, whereas the remaining 56 patients were not treated. The inclusion criteria for the HC group were as follows: (1) aged from 18 to 55 years old; (2) without any present or past significant medical or neurological illness; (3) without history of psychiatric illness in first-degree relatives; (4) without history of head injury; and (5) qualified to undergo an MRI.

### Image Acquisition

All participants underwent MRI scanning using a MAGNETOM Trio Tim 3.0 T system (Siemens Medical Solutions, Germany) equipped with a 32-channel head coil at the Shanxi Provincial People’s Hospital in Taiyuan. None of the participants, identified by two experienced radiologists as having significant brain abnormalities, was excluded from the study. During the scan, all participants were asked to relax with their eyes closed but to remain awake. Using 3D-FLASH sequence, a high-resolution T1-weighted MRI image was acquired with the parameters as follows: repetition time = 2,300 ms, echo time = 2.95 ms, FOV = 225 × 240 mm, matrix = 240 × 256 mm, flip angle = 9°, and voxel size = 0.9375 × 0.9375 × 1.2 mm^3^.

### Voxel-Based Morphometry

To examine differences in GMV among the three groups, VBM analysis was performed using SPM8^1^ and VBM8^2^ toolkits. First, all T1-weighted images were corrected for bias-field inhomogeneity and then segmented into gray matter (GM), white matter, and cerebrospinal fluid. After segmentation, the GM maps were registered into the MNI space using both linear and non-linear affine transformations. Finally, the normalized GM images were smoothed with a Gaussian kernel of 8 mm full width at half maximum for statistical analyses.

### Statistical Analyses

The demographic and clinical characteristics among the UD, BD, and HC groups were compared using SPSS 22.0 version. A one-way analysis of variance (ANOVA) was used to compare the age and years of education for the three groups. The χ^2^se-test was used to compare the sex differences among the groups. Statistical significance was set at *p* < 0.05.

Whole-brain VBM maps of the three groups were compared by ANOVA with total brain volume and sex information as covariates to identify abnormalities among the three groups, and *post hoc* two samples *t*-tests with total brain volume and sex information as covariates were used to identify differences between each pair of groups. The significant level was determined using the AlphaSim correction method with voxel level *P* < 0.001 and cluster level *P* < 0.05. Moreover, the average GMV values of brain areas with changed GMV were extracted, and the correlation analyses between the GMV values and the clinical performances of the UD group and BD group were calculated. The significant level was set at *P* < 0.05 with Bonferroni correction.

## Results

### Demographic and Clinical Comparisons

There was no statistical difference in age, sex, or years of education among the three groups (All *P* > 0.05). Moreover, there was no statistical difference in the HAMD-24, HAMA, and YMRS scores between the BD and UD groups (Table 1).

**TABLE 1:** Demographic and clinical characteristics of all the participants.

	UD group (*n* = 36)	BD-I group (*n* = 22)	HC group (*n* = 27)	Statistical value	*P*-value
Sex (M/F)	16/20	11/11	13/14	0.188	0.910^a^
Age (years)	34.11 ± 10.39	35.17 ± 9.98	32.44 ± 8.57	0.802	0.436^b^
Duration of disease (month)	10.19 ± 5.28	11.14 ± 4.5		0.7	0.49
Education (years)	12.38 ± 3.25	12.10 ± 3.37	13.96 ± 3.24	2.238	0.079^b^
HAMD-24 scores	25.52 ± 4.27	24.45 ± 3.52	N/A	1.424	0.164^c^
HAMA scores	17.76 ± 5.70	17.18 ± 3.82	N/A	0.309	0.759^c^
YMRS scores	1.81 ± 0.82	2.10 ± 0.67	N/A	0.102	0.253^c^

*UD, unipolar depression; BD-I, type I bipolar depression; HC, healthy control; HAMD-24, 24-item Hamilton Depression Rating Scale; HAMA, Hamilton Anxiety Scale; YMRS, Young Mania Rating Scale; M, male; F, female; N/A, not applicable.*

*^*a*^chi-square test; ^*b*^one-way ANOVA; ^*c*^two-sample *t*-test.*

### Voxel-Based Morphometry Analysis

**FIGURE 1:**
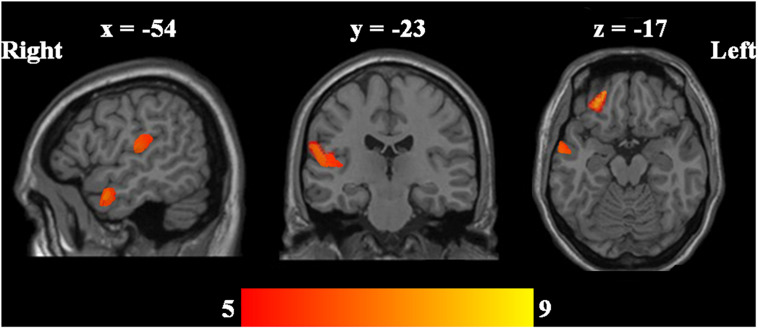
ANOVA was performed to determine whether there are GMV differences among UD, BD, and HCs. Significant level was defined using AlphaSim correction *P* < 0.05 with voxel-level *P* < 0.001.

Using ANOVA, the regional GMV in the left middle temporal gyrus, orbital part of the left middle frontal gyrus, orbital part of the left inferior frontal gyrus, and left supramarginal gyrus showed significant differences among the three groups (Table 2 and Figure 1).

**TABLE 2:** Regional GMV differences between UD groups, BD groups and HC groups.

Brain areas	Cluster size	BA	L/R	MNI coordinates			*F*-value
				x	y	z	(peak)
**Differences among three groups**							
Middle temporal gyrus	245	21	L	−59	2	−20	7.807
Orbital part of the inferior frontal gyrus	401	11/47	L	−27	42	−17	7.774
Supramarginal gyrus	450	40	L	−54	−23	13	7.344
**UD > BD**							
Supramarginal gyrus	484	40	L	−54	−21	15	3.295
**BD < HC**							
Orbital part of the middle frontal gyrus	156	10, 10	L	−24	56	10	−3.351
**UD < HC**							
Orbital part of inferior frontal gyrus	299	11, 11, and 47	L	−27	42	−15	−3.325

*BA, brodmann area; MNI, montreal neurological institute; L/R, left/right hemisphere.*

**FIGURE 2:**
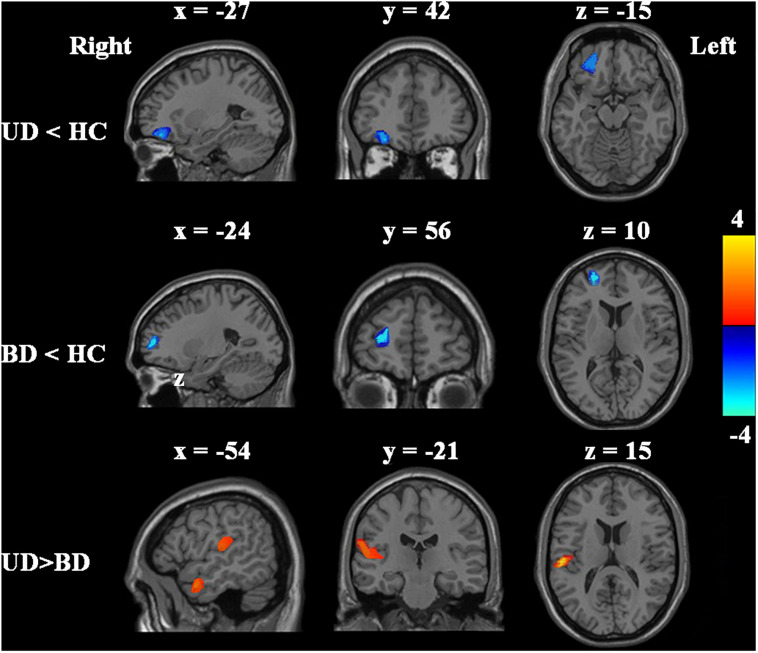
*Post hoc* two-sample *t*-tests were performed to identify GMV differences between UD, BD, and HCs groups. Significant level was determined using AlphaSim correction *P* < 0.05 with voxel-level *P* < 0.001.

Compared with HCs, the UD patients showed significantly decreased GMV in the orbital part of the left inferior frontal gyrus, whereas BD patients showed significantly decreased GMV in the orbital part of the left middle frontal gyrus (Table 2 and Figure 2). Compared with BD patients, UD had increased GMV in the left supramarginal gyrus and middle temporal gyrus (Table 2 and Figure 2).

### Associations Between the Altered Gray Matter Volume and Clinical Characteristics

In the UD and BD groups, Pearson’s correlation analyses were performed between the altered GMV and the HAMD-24 and YMRS scores. There were no significant associations between the GMV and HAMD-24 (*r* = 0.17 and *P* > 0.05) or YMRS (*r* = 0.23 and *P* > 0.05).

## Discussion

In this study, we reported that patients with mood disorders (UD and BD) showed specific GMV differences in the left middle temporal gyrus, orbital part of the middle frontal gyrus, inferior frontal gyrus, and supramarginal gyrus. These findings were consistent with the reports by previous studies (Yang et al., 2017; Kandilarova et al., 2019). The previous studies of UD or BD patients demonstrated reduced GMV in the amygdala, ventral striatum, posterior cingulate cortex, hippocampus, parahippocampal cortex, superior temporal gyrus, and temporopolar cortex (Drevets, 2007; Wang et al., 2017). A previous study about UD found reduced GMV in the temporal lobe regions, especially in the superior temporal gyrus (Ma et al., 2012). Additionally, in young and middle-aged patients with a familial risk of depression, the subjects have a thinner cortex than HCs in the extrahippocampal medial temporal lobe and other brain regions (Peterson et al., 2009). In BD patients, smaller volume and less gray matter densities in the left ventromedial temporal lobe, thinner cingulate, and orbital cortex were found compared with HCs, and changed cortical thickness was associated with abnormal autonomic response to emotional stimuli (Lochhead et al., 2004; Lyoo et al., 2006). All these findings suggested that structural changes may be the neuropathological basis and a neural marker to distinguish the UD and BD patients (Donix et al., 2018).

Gray matter volume differences between UD and BD have been reported in a previous study that found significantly less GMV in the hippocampal formation, insula, and temporal pole in patients with BD than in patients with UD (Redlich et al., 2014). Moreover, Versace et al. (2010) reported that BD showed less fractional anisotropy in the left superior longitudinal fasciculus of the supramarginal gyrus and left posterior cingulum than those with UD (Wise et al., 2016b). In addition, bipolar I disorder patients showed decreased activation in the middle temporal gyrus compared with UD patients (Cerullo et al., 2014). In our study, we further revealed that patients with BD had decreased GMV in the left supramarginal gyrus and middle temporal gyrus than UD patients. Considering these regions play an important role in emotion processing and regulation, we speculated that the above brain regions with structural and functional changes may be the neuroanatomical basis of clinical symptoms of UD and BD. However, we found that the altered GMV was not associated with HAMD-24 scores, suggesting that this difference may be related to the disease itself but not to its clinical symptoms. This is just a speculative explanation and cannot rule out that the clinical symptoms caused by the altered brain regions of UD and BD groups not reflected in the HAMD-24 scores. Therefore, it is necessary to expand the sample size for further research and exploration.

Prefrontal cortex is a complex structure whose main function is related to control emotion, attention, reward processing, and response inhibition (Cerullo et al., 2009). The orbital part of the PFC is located in the rostral frontal lobe. The orbital part of the PFC is involved in learning, memory, higher cognitive function, emotional regulation, and psychiatric disorders (Downar, 2019). Our results showed that UD and BD patients had less GMV in the orbital part of the left middle frontal gyrus and inferior frontal gyrus compared with HCs. This finding was supported by the previous structural and meta-analysis studies that reported decreased cortical thickness and decreased functional activities in UD and BD patients (Fitzgerald et al., 2008; Lim et al., 2013; Han et al., 2014; Abe et al., 2015; Fung et al., 2015). All the results demonstrated that both BD and UD patients have structural changes in the orbital part of the PFC, which may be associated with abnormalities in emotional and cognitive functions.

There are some limitations to this study. First, our study only includes a small sample, and all these findings need to be replicated in larger sample size and a wider range of symptoms to further confirm the experimental results. Second, children and the elderly were not included in the study to exclude the influence of immature children and physical diseases of the elderly on the results. Third, because of some UD and BD patients with high HAMA scores, this may affect our current findings. Fourth, we did not measure the IQ for patients; whether IQ affects the findings needs to be further validated. Finally, a long-term follow-up study is needed to determine whether UD patients convert to BD.

In conclusion, we revealed specific brain structural abnormalities in BD and UD patients using VBM analyses. These findings provided us with the opportunities to identify UD and BD and to gain knowledge of the neural underpinning of affective disorders. The specific differences in GMV found between BD and UD may serve as effective biomarkers to distinguish UD from BD in the future.

## Data Availability Statement

The raw data supporting the conclusions of this article will be made available by the authors, without undue reservation.

## Ethics Statement

The studies involving human participants were reviewed and approved by the First Hospital of Shanxi Medical University. The patients/participants provided their written informed consent to participate in this study.

## Author Contributions

YX and NS designed the experiments. JW, PL, JW, AZ, SL, and CY performed the clinical data collection and assessment. JW and PL performed the neuroimaging data analysis and wrote the draft. All the authors discussed the results and reviewed the manuscript.

## Conflict of Interest

The authors declare that the research was conducted in the absence of any commercial or financial relationships that could be construed as a potential conflict of interest.

## Footnotes

1 http://www.fil.ion.ucl.ac.uk/spm

2 http://dbm.neuro.uni-jena.de/vbm.html
